# Connective tissue growth factor contributes to joint homeostasis and osteoarthritis severity by controlling the matrix sequestration and activation of latent TGFβ

**DOI:** 10.1136/annrheumdis-2018-212964

**Published:** 2018-06-20

**Authors:** Xiaodi Tang, Hayat Muhammad, Celia McLean, Jadwiga Miotla-Zarebska, Jacob Fleming, Athanasios Didangelos, Patrik Önnerfjord, Andrew Leask, Jeremy Saklatvala, Tonia L Vincent

**Affiliations:** 1 Kennedy Institute of Rheumatology, University of Oxford, Oxford, UK; 2 Department of Clinical Sciences, University of Lund, Lund, Sweden; 3 Department of Dentistry, University of Western Ontario, London, Ontario, Canada

**Keywords:** osteoarthritis, arthritis, chondrocytes

## Abstract

**Objectives:**

One mechanism by which cartilage responds to mechanical load is by releasing heparin-bound growth factors from the pericellular matrix (PCM). By proteomic analysis of the PCM, we identified connective tissue growth factor (CTGF) and here investigate its function and mechanism of action.

**Methods:**

Recombinant CTGF (rCTGF) was used to stimulate human chondrocytes for microarray analysis. Endogenous CTGF was investigated by in vitro binding assays and confocal microscopy. Its release from cut cartilage (injury CM) was analysed by Western blot under reducing and non-reducing conditions. A postnatal, conditional Ctgf^cKO^ mouse was generated for cartilage injury experiments and to explore the course of osteoarthritis (OA) by destabilisation of the medial meniscus. siRNA knockdown was performed on isolated human chondrocytes.

**Results:**

The biological responses of rCTGF were TGFβ dependent. CTGF displaced latent TGFβ from cartilage and both were released on cartilage injury. CTGF and latent TGFβ migrated as a single high molecular weight band under non-reducing conditions, suggesting that they were in a covalent (disulfide) complex. This was confirmed by immunoprecipitation. Using Ctgf^cKO^ mice, CTGF was required for sequestration of latent TGFβ in the matrix and activation of the latent complex at the cell surface through TGFβR3. In vivo deletion of CTGF increased the thickness of the articular cartilage and protected mice from OA.

**Conclusions:**

CTGF is a latent TGFβ binding protein that controls the matrix sequestration and activation of TGFβ in cartilage. Deletion of CTGF in vivo caused a paradoxical increase in Smad2 phosphorylation resulting in thicker cartilage that was protected from OA.

## Introduction

Articular cartilage is an avascular, non-elastic connective tissue in which chondrocytes, the only cells in the tissue, are embedded in a type II collagen and proteoglycan dense matrix. This part of the matrix is designed to withstand mechanical stress and inability to do so can lead to joint failure and osteoarthritis (OA).^1^ Individual chondrocytes are also surrounded by a discrete pericellular matrix (PCM) that is structurally distinct from the adjacent type II collagen-rich matrix.^2^ The PCM is rich in the heparan sulfate proteoglycan, perlecan and type VI collagen.^3 4^ Reduced stiffness of the PCM compared with the adjacent type II collagen rich matrix suggests that this region will compress preferentially on mechanical load.^5^ One mechanism by which cells of cartilage respond to mechanical stress is by release of sequestered heparin-bound molecules, such as FGF2, from the PCM.^6 7^ The mechanism for this release may be due to a rapid flux in sodium that is displaced from the highly sulfated aggrecan-rich matrix on tissue compression. Release of FGF2 drives an immediate injury response in chondrocytes and protects animals from development of OA.^8 9^

TGFβ is another important cartilage growth factor that controls chondrogenesis and contributes to OA pathology.^10–12^ TGFβ is secreted from cells in a latent complex in which a covalent dimer of active TGFβ is non-covalently associated with two latency-associated peptides (LAPs) to form a small latent complex (SLC).^13^ In most cell types, the SLC covalently associates with one of four described latent TGFβ binding proteins (LTBPs) to form a large latent complex (LLC).^14^ They exhibit a range of functions including facilitating folding and secretion and sequestration of the LLC, and activation of latent TGFβ. A number of mechanisms for latent TGFβ activation have been proposed, including integrin-dependent activation in response to mechanical stress,^15–17^ protease-dependent mechanisms^18–20^ and those mediated by thrombospondin.^21^ Genetic manipulation in mice and identification of human mutations in TGFβ ligands, receptors and the LTBPs demonstrate the collective importance of these molecules in many aspects of tissue biology. The modest overlap in the phenotypes suggests that there are temporal and tissue-specific roles for these molecules, and raises the possibility that alternative mechanisms of TGFβ activation exist.^22–26^

In this study, we describe the search for other sequestered molecules of the PCM that are released on cartilage injury. Using a proteomic analysis, we identify connective tissue growth factor (CTGF, also known as CCN2) and determine its function and role in OA development.

### Materials and methods

#### Reagents

See online supplementary file.

10.1136/annrheumdis-2018-212964.supp1 Supplementary data

### Mice

The *Ctgf^fl/fl^* line was developed by AL.^27^ The *Ubi*-Cre/ER^T2^ line was purchased from Jackson Laboratories (strain no. 007001). Gene deletion was induced at week 4 of age (for avulsion hip injury) (men and women) and 8 weeks (for in vivo knee joint studies) (men only) with three intraperitoneal injections of tamoxifen on three consecutive days (50 mg/kg).

**Cartilage and isolation of chondrocytes, confocal microscopy, siRNA transfection, microarray and RT-PCR**, c**o-immunoprecipitation, ELISA-based binding assay for CTGF and perlecan, TGFβ1 ELISA:** See online supplementary methods.

### Statistical analysis

Paired Student’s t-tests were performed when comparing the same cell population with two different treatments. Unpaired t-tests were performed when comparing groups of mice. Not significant (ns), p≤0.05 (*), p≤0.01 (**), p≤0.001 (***).

## Results

### CTGF is a cartilage PCM protein

Four known heparin-binding growth factors were identified by proteomic analysis of purified PCM from human articular cartilage. These included FGF2, CTGF, hepatoma-derived growth factor and CCN1, also known as Cyr61 (data not shown). CTGF was of particular interest because *Ctgf* ^−/−^ mice have a severe musculoskeletal and vascular phenotype resulting in perinatal lethality^28–30^ and the mechanism for this is unexplained.

**Figure 1:**
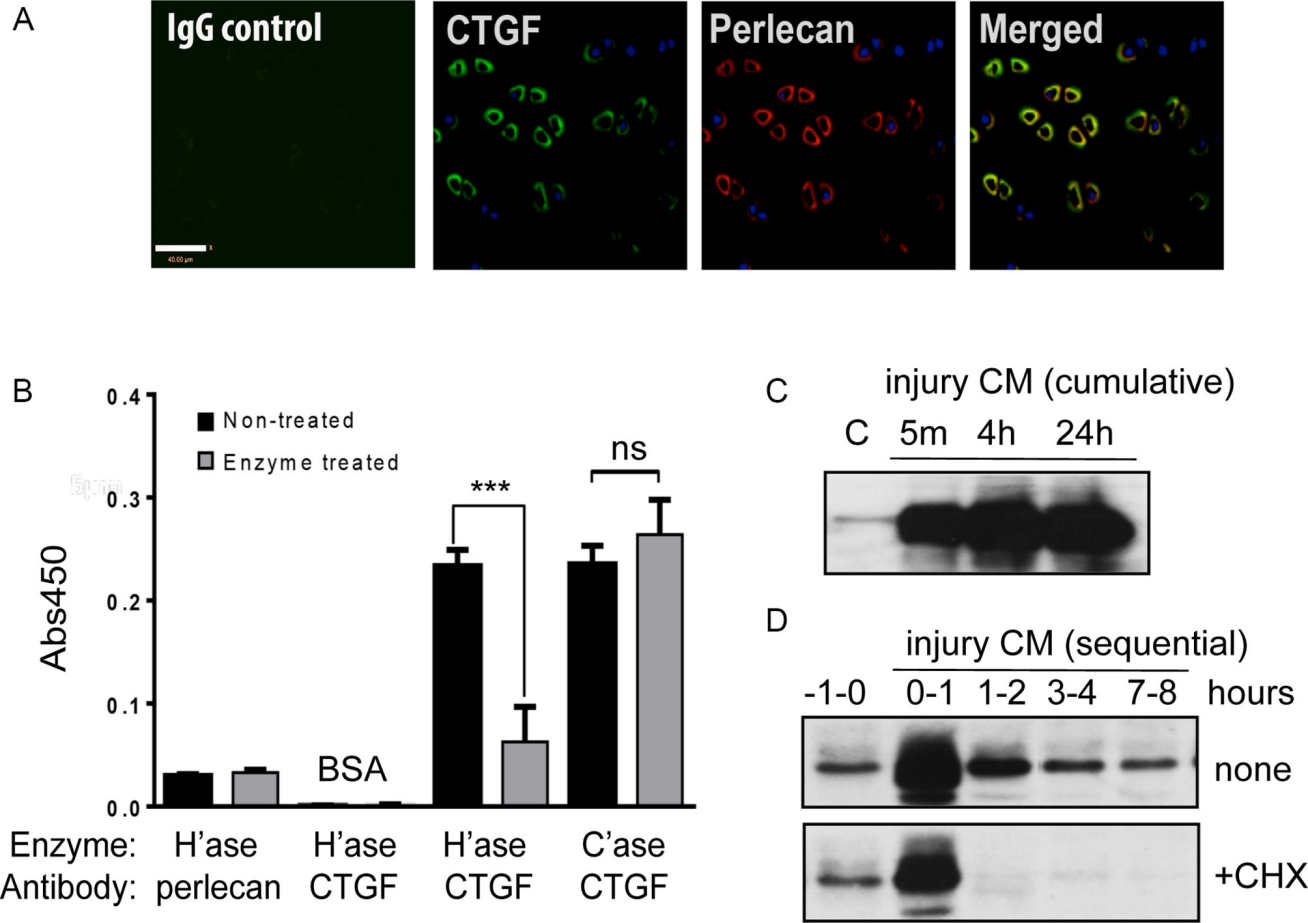
Connective tissue growth factor (CTGF) binds to perlecan in the pericellular matrix of articular cartilage and is released rapidly on injury. (A) Confocal microscopy of normal human articular cartilage, showing pericellular colocalisation of CTGF (green) and perlecan (red). Scale bar, 40 µm. (B) bovine serum albumin (BSA) or perlecan was precoated onto ELISA plates and wells were treated with or without 10 mU/mL of heparitinase (H’ase) or chondroitinase (C’ase) prior to incubation with 0.05 µg recombinant CTGF for 3 hours. CTGF was detected with anti-CTGF antibodies using a standard ELISA plate reader. Levels of bound perlecan pre-enzyme and post-enzyme treatment were checked with an anti-perlecan antibody. (C, D) Porcine articular cartilage explants (5×4 mm discs) were rested in serum-free (SF) medium for 48 hours and re-cut in fresh SF medium in the presence or absence of 10 µg/mL cycloheximide (+CHX). Injury conditioned medium (CM) was collected cumulatively (C) or sequentially (D) after specific time points and immunoblotted for CTGF. ***P<0.001; ns, not significant by a two-sided Student’s t*-*test.

Confocal microscopy confirmed pericellular localisation of CTGF in normal human articular cartilage (figure 1A). Binding of CTGF to perlecan was detected in vitro, in a heparan sulfate-dependent manner (figure 1B). Like FGF2,^6^ CTGF was rapidly released into the medium of injured cartilage (injury CM) (figure 1C). Sequential collection of injury CM after cutting demonstrated that most protein was released in a single burst (figure 1D, none). Subsequent slow accumulation was from an actively translated and secreted pool as it could be inhibited by cycloheximide (figure 1D, +CHX).

### CTGF activates chondrocytes in a TGFβ-dependent manner

**Figure 2:**
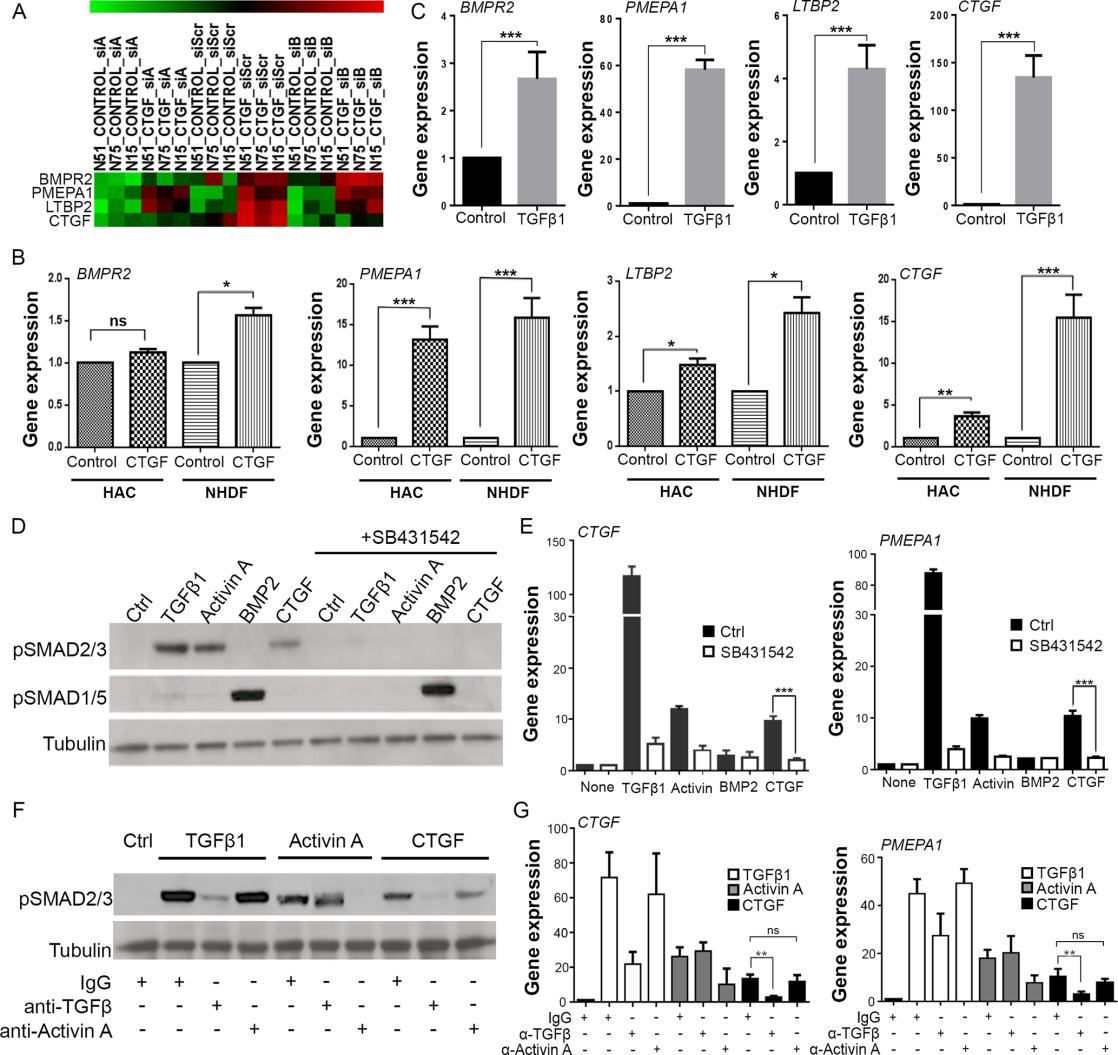
Connective tissue growth factor (CTGF) induces TGFβ-dependent SMAD2 phosphorylation and gene regulation. (A) Heat map of microarray *Z*-scores for the genes upregulated in human chondrocytes 8 hours after stimulation with 100 ng/mL CTGF (in triplicate). siRNAs targeting CTGF (siA, siB) were used to silence endogenous Ctgf prior to stimulation with recombinant ligand. The scrambled siRNA (siScr) is also shown. (B) RT-PCR validation of CTGF-induced genes in human articular chondrocytes (HAC) and normal human dermal fibroblasts (NHDF). (C) RT-PCR of NHDF treated with 10 ng/mL TGFβ1. All gene expressions expressed relative to GAPDH. Porcine chondrocytes were stimulated for either 45 min (D) or 8 hours (E) with 10 ng/mL TGFβ1, 100 ng/mL activin A, 100 ng/mL BMP2 or 100 ng/mL CTGF in the presence or absence of 5 µM SB431542. Lysates were immunoblotted for pSMAD2 and pSMAD1/5 (D), or RT-PCR performed for expression of *CTGF* and *PMEPA1* (E). (F, G) Porcine chondrocytes treated with TGFβ1, activin A or CTGF as above in the presence of 1 µg/mL anti-TGFβ or activin A neutralising antibodies. 45 min lysates were immunoblotted for pSMAD2 (F), or 8-hour RT-PCR performed for expression of CTGF and PMEPA1 (G). Western blots are representative of three independent experiments. All error bars represent SE. *p<0.05, **p<0.01, ***p<0.001 by a two-sided Student’s t-test. ns, not significant. n=3.

To investigate the role of CTGF in articular chondrocytes, we performed a microarray analysis of isolated human articular chondrocytes stimulated with recombinant CTGF. To take into account endogenous production of CTGF in chondrocytes, we silenced endogenous CTGF by siRNA. Four CTGF-induced genes were identified: BMP receptor 2 (*BMPR2*), Prostate Transmembrane Protein, Androgen Induced 1 (*PMEPA1*), latent TGFβ binding protein 2 (*LTBP2*) and CTGF itself (*CTGF*) (figure 2A). Apart from *BMPR2*, regulation of each of these was robust in CTGF-stimulated human dermal fibroblasts and human articular chondrocytes by RT-PCR (figure 2B) even though the responses were slightly less strong in chondrocytes. The four CTGF-induced genes were known to be TGFβ-responsive genes, which we confirmed by RT-PCR in human dermal fibroblasts (figure 2C). We checked to see whether recombinant CTGF was able to activate the canonical pathway of TGFβ involving phosphorylation of SMAD2. Phosphorylation of SMAD2, but not SMAD1/5/8, occurred following TGFβ, activin A (a family member of TGFβ that signals through the ALK4 receptor, also known as ACVR1B) or CTGF stimulation (figure 2D). Phosphorylation by all three ligands was abrogated in the presence of the ALK4/5/7 receptor inhibitor (figure 2D, SB431542), as was the gene regulation of *CTGF* and *PMEPA1* by these ligands (figure 2E). CTGF did not stimulate the TGFβ receptor directly, as phosphorylation of SMAD2 by CTGF was inhibited by a neutralising antibody to TGFβ (figure 2F), as was CTGF-induced gene regulation (figure 2G). SMAD2 phosphorylation and gene regulation by CTGF was not abrogated by an activin A neutralising antibody (figure 2F, G).

We checked that there was no biologically significant contamination of TGFβ in our purified CTGF preparation (<0.1 ng TGFβ/100 ng CTGF) (data not shown), and we were unable to demonstrate synergy between suboptimal doses of recombinant TGFβ and CTGF in isolated cells (data not shown).

### CTGF is secreted and sequestered in the PCM in a covalent complex with latent TGFβ

**Figure 3:**
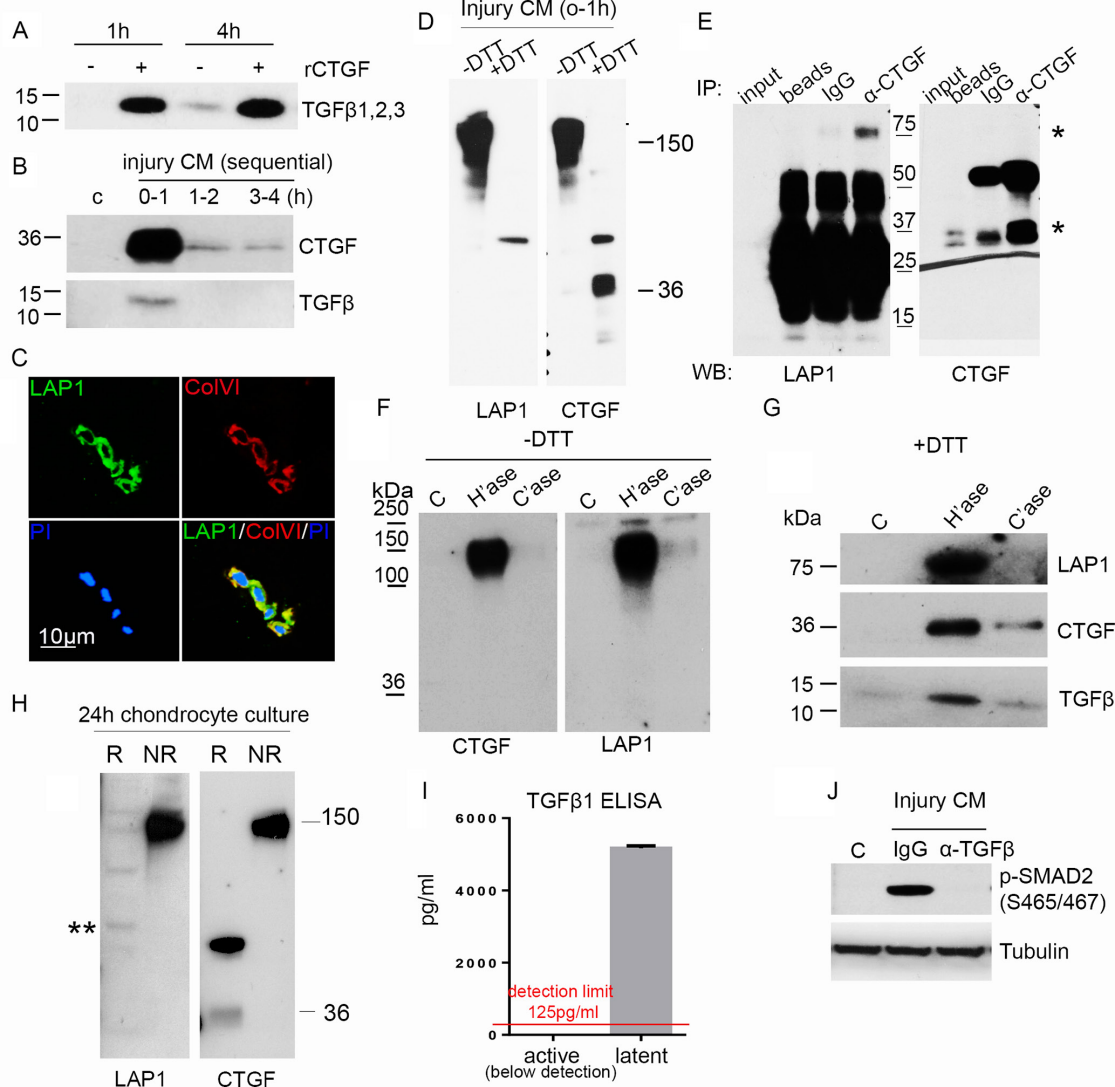
Connective tissue growth factor (CTGF) is covalently bound to latent TGFβ and is sequestered in the pericellular matrix (PCM) on heparan sulfate. (A) Rested porcine articular cartilage was treated with 100 ng/mL CTGF for 1 hour or 4 hours and the medium immunoblotted (under reducing conditions) for TGFβ. (B) Rested porcine cartilage was re-cut in fresh medium and the injury CM, at times specified, immunoblotted for CTGF and TGFβ (under reducing conditions). (C) Confocal microscopic images showing LAP1 and type VI collagen (ColVI) colocalising in the PCM of normal human articular cartilage, propidium iodide (PI). Scale bar, 10 µm. (D) Cartilage injury CM (1 hour) was electrophoresed under both reducing (+DTT) or non-reducing (−DTT) conditions and immunoblotted for LAP1 and CTGF. (E) CTGF was immunoprecipitated from injury CM using goat anti-CTGF antibody and immunoblotted for LAP1 (first panel) or CTGF (second panel) (under reducing conditions). * indicating bands for LAP1 and CTGF. (F) Cartilage explants were treated with or without 10 mU/mL heparitinase or chondroitinase for 4 hours. Medium was run under either non-reducing (−DTT) (F) or reducing (+DTT) (G) conditions and immunoblotted for LAP1, CTGF and TGFβ. (H) Culture medium was collected from isolated monolayer porcine chondrocytes over 24 hours and immunoblotted for CTGF or LAP under reducing or non-reducing conditions. ** shows weak 75 kDa band of LAP1. (I) Injury CM was treated with or without hydrochloric acid to calculate active and latent TGFβ1 protein levels by ELISA. Lower limit of detection, 125 pg/mL (n=3). (J) Porcine chondrocytes were treated (45 min) with or without injury CM pre-incubated with 1 µg/mL anti-TGFβ neutralising antibody or isotype control for 1 hour. Lysates were immunoblotted for pSMAD2 and tubulin.

CTGF did not induce mRNA for TGFβ (data not shown), but as TGFβ-dependent activity was increased by CTGF, we next investigated whether CTGF controlled TGFβ protein levels. Stimulation of articular cartilage explants with recombinant CTGF led to strong accumulation of TGFβ protein in the medium within 1 hour of stimulation (figure 3A). Moreover, endogenous TGFβ and CTGF were detected in the medium following simple cutting injury within 1 hour (figure 3B). The rapid release of TGFβ from injured cartilage suggested that it was also in a pre-formed sequestered store. We determined TGFβ was stored in an extracellular pool by demonstrating staining for the latency-associated protein (LAP1) of latent TGFβ in the PCM (colocalising with type VI collagen) (figure 3C).

As latent TGFβ usually exists as a complex in which there are several disulfide bonds, injury CM was separated by SDS-PAGE under non-reducing and reducing conditions and immunoblotted for LAP1 and CTGF. Surprisingly, LAP1 and CTGF co-migrated at 150 kDa (figure 3D), and immunoprecipitation of CTGF pulled down LAP (figure 3E, band at 75 kDa) indicating that CTGF and latent TGFβ were in a covalent (disulfide) complex. These results were strengthened further by showing that treatment of cartilage with heparitinase led to release of the 150 kDa CTGF/LAP complex (figure 3F), which, when run under reduced conditions, contained both CTGF (at 37 kDa) and components of the small latent complex (LAP1 and TGFβ) (figure 3G). Having established that CTGF was covalently bound to latent TGFβ in the extracellular matrix, we determined what fraction of secreted CTGF was bound to latent TGFβ. Examining the non-reduced 24-hour culture medium from isolated chondrocytes, all detectable CTGF co-migrated with LAP as a single high molecular weight band at 150 kDa, suggesting that CTGF’s principal role in chondrocytes is as a latent TGFβ binding protein (figure 3H, PACs). In this experiment, both CTGF and LAP1 failed to be reduced fully; CTGF was seen at 36 kDa (monomeric form) as well as migrating at 70 kDa. LAP1 migrated at several molecular weights including its predicted fully reduced form, 75 kDa (asterisk).

### CTGF is required for activation of latent TGFβ

We next investigated whether TGFβ released from the PCM on injury was all in its latent form or whether injury also caused activation of the latent complex. Free TGFβ was not detected in the injury CM by ELISA (figure 3I). However, chondrocytes stimulated with injury CM showed strong TGFβ-dependent SMAD2 activity (figure 3J), suggesting that the complex is stored and released in its latent form then activated on contact with the cell. We were never able to detect free LAP or CTGF in the medium of these stimulated cells indicating that these are rapidly cleared (most likely through an endocytic pathway).

**Figure 4:**
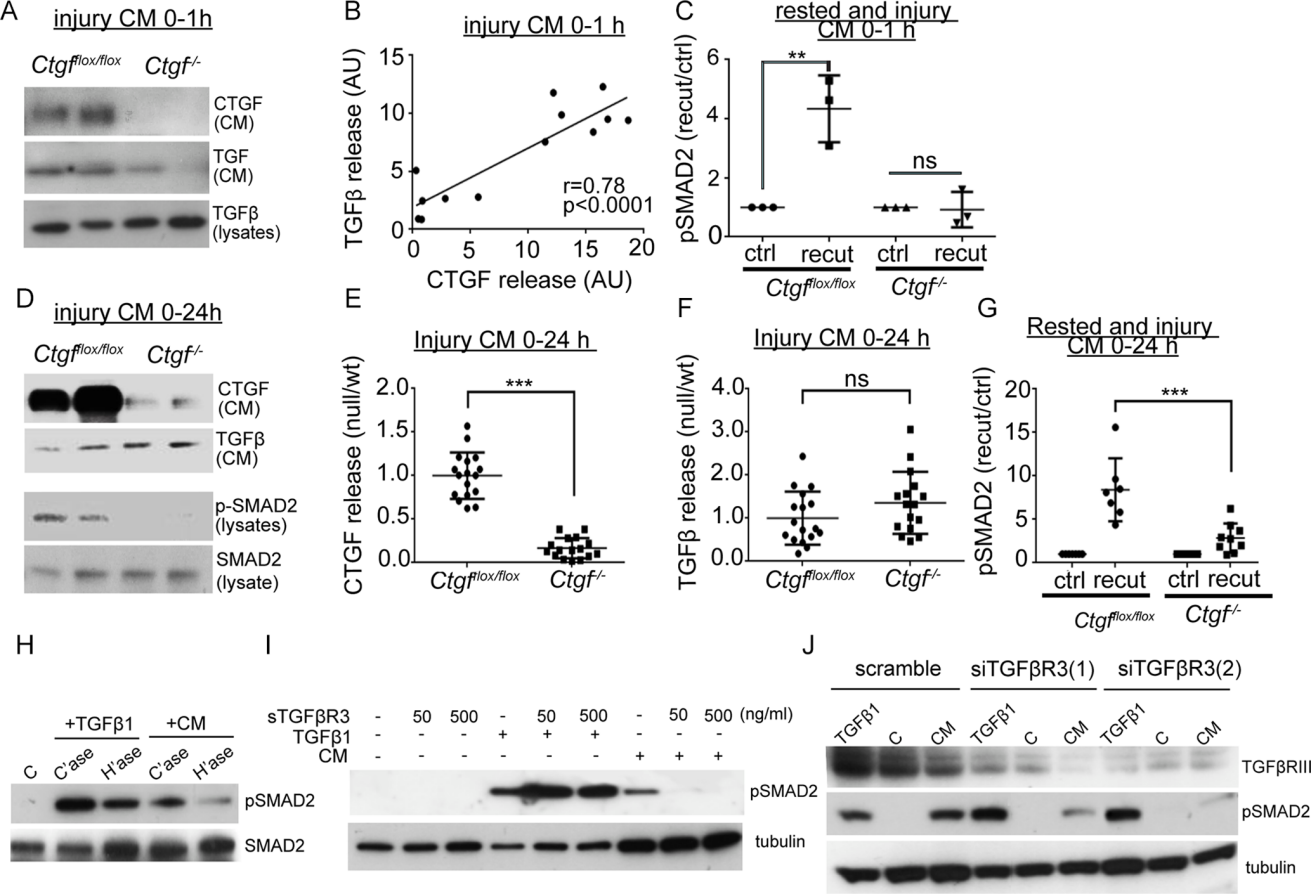
Connective tissue growth factor (CTGF) is required for release and activation of latent TGFβ in a TGFβR3-dependent manner. Conditional, inducible deletion of CTGF (Ctgf^cKO^) was achieved by crossing Ctgf^fl/fl^ (wt) mice with an inducible Cre recombinase driven by the ubiquitin promoter (*UbiCreER* ^T2^). Mice were treated with tamoxifen at 4 weeks to induce deletion. Hip avulsion, a model of murine cartilage injury, was performed in 6-week-old Ctgf^cKO^ or wt mice. Injury medium (serum free) was conditioned for either 1 hour (A–C) or 24 hours (D–G). Some medium was conditioned from hips that had been rested for 48 hours then re-cut to obtain control (ctrl) and re-cut injury medium. (A) Injury CM or explant lysates were immunoblotted for CTGF and TGFβ (run under reducing conditions). (B) Protein levels of injury CM CTGF and TGFβ were quantified (wt, n=7; null, n=6) and their correlation examined. (C) Control and injury CM was used to stimulate porcine chondrocytes (45 min) and lysates immunoblotted for pSMAD2. (D) Injury CM (24 hours) was generated from Ctgf^cKO^ and wt hips and were immunoblotted for CTGF and TGFβ (run under reducing conditions). Injury CM was also used to stimulate monolayer chondrocytes for 45 min and the lysates were immunoblotted for pSMAD2 (lower panels). Released CTGF (E) and TGFβ (F) from the injury CM were quantified (wt, n=17; null, n=16) and expressed relative to wt levels. (G) Injury CM was used to stimulate porcine chondrocytes (45 min) and pSMAD2 was quantified from Western blots. (H–J) To determine the mechanism of activation of latent injury CM, porcine chondrocytes were stimulated with either injury CM or TGFβ with the following pre-treatments: (H) 10 mU/mL heparitinase or chondroitinase for 4 hours prior to stimulation. (**I**) 50 ng/mL or 500 ng/mL soluble TGFβR3, 1 hour prior to stimulation. (J) TGFβR3 was knocked down by siRNA for 72 hours and the cells were rested in serum-free dulbecco modified eagles medium (DMEM) for 18 hours prior to stimulation. Error bars represent SE. **p<0.01, ***p<0.001 by a two-sided Student’s t-test; ns, not significant. Pearson’s coefficients of linear correlation and p values are shown.

To determine whether CTGF was required for controlling release of the latent complex on injury and its activation at the cell surface, we generated mice in which CTGF had been deleted ubiquitously in an inducible (postnatal) manner (*Ctgf^fl/fl^*/*UbiCreERT^2^).* Successful deletion was confirmed by showing reduced release of CTGF from knockout hip cartilage in the first hour following injury compared with wild-type hips (figure 4A). TGFβ release within the first hour of hip injury was also significantly reduced and correlated with the level of CTGF in the injury medium suggesting that CTGF is required for the release of TGFβ on cartilage injury (figure 4A,B). In the absence of both CTGF and TGFβ, the 1 hour injury CM from *Ctgf^cKO^* hips was unable to activate SMAD2 in isolated chondrocytes (figure 4C). When *Ctgf^vKO^* and wild-type cartilage was cultured for 24 hours following injury, CTGF levels remained suppressed in *Ctgf^cKO^* 24-hour CM (figure 4D,E), but TGFβ accumulated in the medium (due to constitutive secretion of the SLC by the chondrocytes over this time) (figure 4D,F). Despite the presence of TGFβ, the 24-hour injury CM from *Ctgf^cKO^* hips was unable to phosphorylate SMAD2 in isolated porcine chondrocytes (figure 4D lower panel, 4G), and the ability of the 24-hour injury CM to phosphorylate SMAD2 strongly correlated with levels of CTGF (r=0.80, p=0.0085) but not with TGFβ (r=0.42, p=0.151).

### Activation of the latent CTGF–TGFβ complex requires CTGF binding to cell surface TGFβR3 in a heparan sulfate-dependent manner

We speculated that the CTGF-bound TGFβ complex was binding to a cell surface receptor to allow activation of latent TGFβ. Published mechanisms for activation of latent TGFβ in other tissues point towards a role for integrin binding or metalloproteinase activity.^31 32^ To establish whether cell surface integrins were involved in CTGF-dependent SMAD2 activation by the injury CM, we stimulated human chondrocytes after pretreatment with the soluble arginylglycylaspartic acid (RGD) peptide (to block integrin binding) or with neutralising antibodies to αv, β1 or β3 integrins. None of these approaches affected SMAD2 activation by the injury CM (online supplementary figure 1a). Nor was activity affected by preincubation with a pan-metalloproteinase inhibitor, GM6001 (online supplementary figure 1a). As CTGF is known to bind and be cleared from the extracellular space by the scavenger receptor low density lipoprotein receptor-related protein 1 (LRP1), we treated cells with receptor-associated protein (RAP), an inhibitor of LRP1 re-uptake, or knocked down LRP1 by siRNA. Neither of these approaches affected activity of the injury CM (online supplementary figure 1b,c).

10.1136/annrheumdis-2018-212964.supp3 Supplementary data

Finally, we addressed whether activation of the injury CM was dependent on cell surface heparan sulfate. Treatment of isolated chondrocytes with heparitinase, but not chondroitinase, prior to stimulation with the injury CM significantly blunted activation of SMAD2 (figure 4H). One transmembrane heparan sulfate proteoglycan that has been described as a regulator of TGFβ signalling (but not latent TGFβ activation) is betaglycan, also known as TGFβR3.^33 34^ Soluble TGFβR3 was able to abrogate injury CM-induced activation of SMAD2 (figure 4I) and activity was also suppressed following knockdown of TGFβR3 using two separate siRNA oligonucleotides (figure 4J).

### CTGF deletion causes a paradoxical hyper-Smad2 phosphorylation and protects cartilage from OA

**Figure 5:**
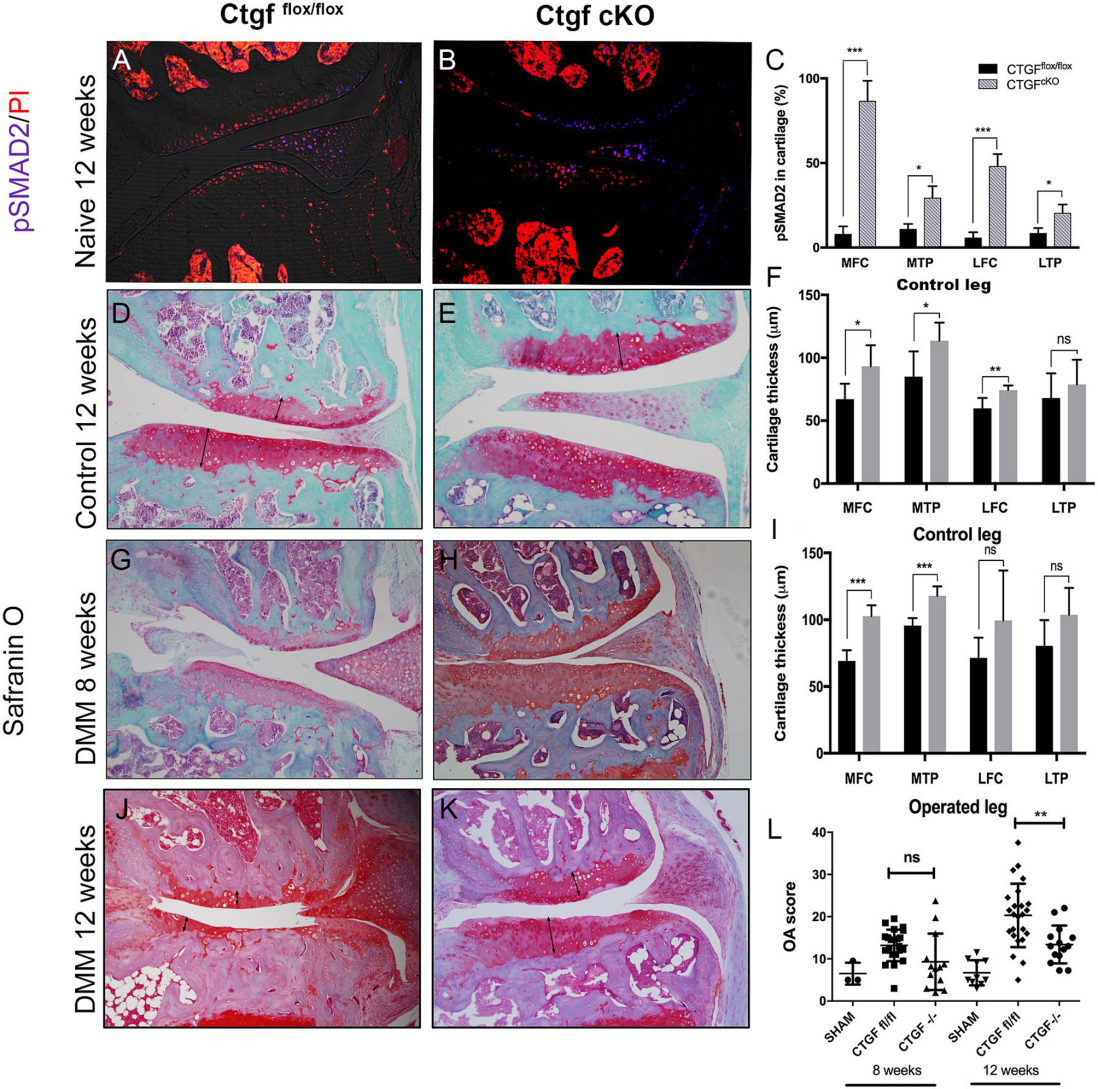
Paradoxical increase in SMAD2 phosphorylation in cartilage of CTGF^−/−^ mice is associated with thicker cartilage and protection against osteoarthritis (OA). Male Ctgf^fl/fl^;UbiCreER^T2^ and Ctgf^fl/fl^ (control) mice were treated with tamoxifen at 8 weeks of age to induce postnatal, pan-tissue deletion of connective tissue growth factor (CTGF^cKO^). At 10 weeks, surgical destabilisation of the joint, by cutting the meniscotibial ligament (DMM), or sham surgery, was performed on the right knee joint. The contralateral limb was used as a control. Joints were examined histologically 8 and 12 weeks postsurgery. Cartilage degradation (OA score) and cartilage thickness measures were performed on Safranin O-stained sections. Immunohistochemistry was performed for phospho-SMAD2. Magnification ×20.

To assess the role of CTGF in vivo, *Ctgf^fl/fl^*/*UbiCreERT^2^* male mice were treated with tamoxifen at 8 weeks of age to induce deletion of CTGF and the joints examined 10 weeks later. No overt ill health was observed in these mice. Surprisingly, deletion of Ctgf was associated with markedly increased phosphorylation of SMAD2 in the chondrocytes across all compartments of the joint (figure 5A–C) and the articular cartilage was significantly thicker in the Ctgf^cKO^ control mice (figure 5D–F). Joint destabilisation was performed at 10 weeks of age and histomorphometry of the operated and control (contralateral) joints was performed. The thicker cartilage of the *Ctgf* ^cKO^ mice was more resistant to degradation induced by surgical joint destabilisation (figure 5G–L). Osteophyte size and maturity were not affected by genotype (online supplementary figure 2c).

10.1136/annrheumdis-2018-212964.supp4 Supplementary data

### SMAD2 phosphorylation of the articular cartilage following CTGF deletion may be due to compensatory regulation of TGFβ ligands from other tissues of the joint

In order to explore the paradoxical increase in SMAD2 phosphorylation in the cartilage of Ctgf^cKO^ mice, we extracted mRNA either from cartilage (auricular) or the whole joint of mice 2 weeks following tamoxifen treatment. A total of 38 genes relating to TGFβ, including ligands, receptors and target responses, were investigated. Gene regulation was expressed relative to wild-type tissue (online supplementary table 1). The results confirmed knockdown of CTGF in both cartilage and whole joints (97% and 87%, respectively). When the cartilage was considered separately, a small number of genes were regulated; these included a statistically significant reduction in follistatin, BMP6, aggrecan and LTBP2, and a striking increase in type II collagen (approaching threefold). This was quite different to that observed in the whole joint where a number of TGFβ family members were increased (inhibin βA, TGFβ2, BMP7) and regulators of these pathways (TGFβR1, Acvrl1, Bmpr2, Bmpr1a, Smurf1).

10.1136/annrheumdis-2018-212964.supp2 Supplementary data

## Discussion

Here, we describe CTGF as a novel latent TGFβ binding protein, binding covalently to the small latent complex of TGFβ prior to secretion, sequestering latent TGFβ in the matrix of cartilage in a heparan sulfate-dependent manner and controlling its release on cartilage injury. Activation of the complex in chondrocytes occurs exclusively in a CTGF-dependent and TGFβR3-dependent manner. It may also explain how cells that do not express α_v_β_6_, an important latent TGFβ-activating integrin,^15 16^ are able to activate the latent growth factor.

There is a strong existing literature to support a link between CTGF and TGFβ. Although some publications point towards a synergistic relationship between the two cytokines, the mechanism by which CTGF influences TGFβ has remained controversial.^35–37^ In vitro, fragments of CTGF have been shown to bind directly to recombinant (active) TGFβ to activate the TGFβ receptor synergistically.^38^ However, other diverse mechanisms of cellular activation by CTGF have been described including through extracellular integrin engagement,^30^ and binding to cell surface receptors, TRK-A^39^ and LRP1.^40^ We found no evidence for activation of CTGF by these mechanisms. Most studies presented in our study were performed on endogenously secreted and released CTGF, which likely explains why we uncovered this novel mechanism of action. Our ability to demonstrate a cellular response with recombinant protein in chondrocytes (figure 2) was most likely due to displacement of endogenous extracellular CTGF–TGFβ from the chondrocyte cultures.

**Figure 6:**
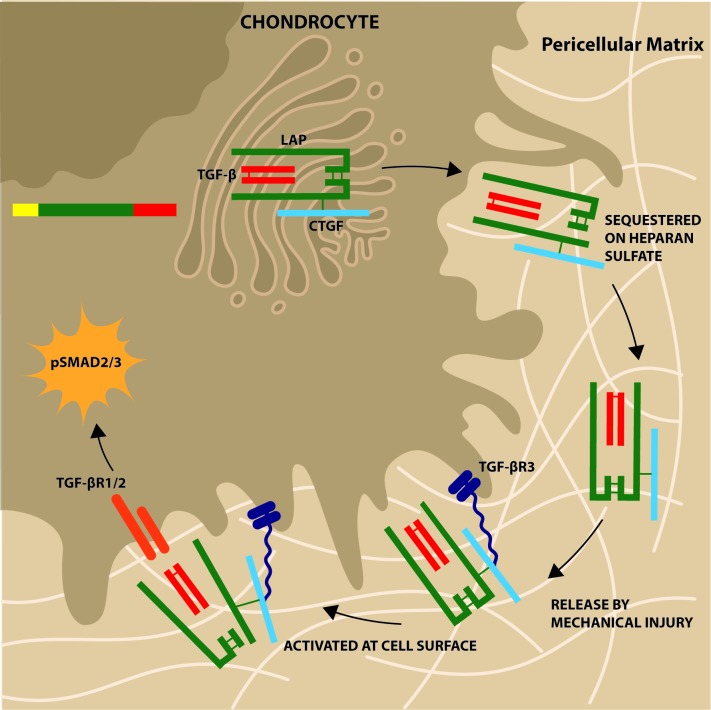
Schematic of role of connective tissue growth factor (CTGF) in cartilage. CTGF is covalently bound (disulfide bond) to latent TGFβ in the endoplasmic reticulum of the chondrocyte. It is secreted as a large latent complex and sequestered in the pericellular matrix attached to the heparan sulfate chains of perlecan. Mechanical compression causes release of heparan sulfate bound factors (mechanism likely involving sodium flux) and latent complex engages with TGFβR3 on cell surface (in heparan sulfate/CTGF-dependent manner). This allows activation of latent complex with autocrine activation of canonical pathway of TGFβ involving SMAD2/3 phosphorylation.

The role of TGFβR3 in the activation of latent TGFβ has not been described before. Active TGFβ is known to bind to the core protein of TGFβR3 in a glycosaminoglycan-independent fashion where it enhances TGFβ signalling.^33^ TGFβR3 does not have the ability to signal directly as it has only a short cytoplasmic tail, but the N-terminal region is thought to interact with the TGFβ type II receptor and thereby facilitate recruitment of the receptor complex and TGFβ-induced SMAD2 phosphoryation.^41^ In renal mesangial cells, active ligand binds to TGFβR3 to antagonise signalling.^42^ In chondrocytes, we observed that TGFβR3-dependent activation of latent TGFβ required heparan sulfate (figure 4h). As we demonstrated that CTGF binds to heparan sulfate (on perlecan) in vitro and within the PCM, we hypothesise that it is a CTGF–heparan sulfate interaction that mediates initial binding of the latent complex to TGFβR3 (figure 6). Thereafter, we propose that this facilitates activation of latent TGFβ, through a mechanism not yet understood, but not involving integrin ligation or metalloproteinase activity, to allow it to activate the adjacent TGFβR1/2 receptor complex.

TGFβ is regarded as a chondroprotective agent in articular cartilage, promoting chondrogenesis in mesenchymal stem cells and inhibiting terminal differentiation.^43 44^ Similar biological effects have been described for CTGF.^45 46^ Canonical TGFβ signalling is through phosphorylation of SMAD2/3, leading to the activation and nuclear translocation of SMAD4. Human mutations in SMAD3 or deletion of SMAD3 in mice is associated with increased risk of osteoarthritis.^43 47 48^ Although we did not examine SMAD3 phosphorylation directly, SMAD2 was phosphorylated both in vitro after stimulation with TGFβ or CTGF and, somewhat paradoxically, in vivo after CTGF deletion. This was associated with thicker cartilage, which was more resistant to degradation. SMAD2/3 phosphorylation also occurs after stimulation with other members of the TGFβ family such as nodal and activin βA. In our RNA analysis of CTGF cKO joints, we found no detectable nodal expression, but we did find an increase in mRNA expression of inhibin βA (the dimer of which forms activin βA), as well as an increase in TGFβ2. Although it could be that these effects are due to CTGF deletion in the chondrocytes, when the cartilage was considered separately, these ligands did not appear increased raising the possibility that pan-deletion of CTGF is leading to increased activin and TGFβ synthesis from other tissues of the joint. Interestingly, a similar paradox is documented in patients with Loeys-Dietz syndrome where loss of function of TGFβ (through mutations in TGFβ receptors or ligands) is associated with unexplained high SMAD2 phosphorylation in patient tissues.^49^ Another possible explanation for the increased SMAD2 phosphorylation in the chondrocytes of CTGF^cKO^ mice is that there is increased soluble latent TGFβ either because it is not being sequestered in the matrix of cartilage or because it is derived from other cells of the joint. The chondrocytes may, under these circumstances, be able to compensate for the loss of CTGF by activating latent TGFβ in a CTGF-independent and TGFβR3-independent manner. Whether this is through an LTBP-dependent mechanism remains unclear.
