# Supplementary material for: Connective tissue growth factor contributes to joint homeostasis and osteoarthritis severity by controlling the matrix sequestration and activation of latent TGFβ
Source: Ann Rheum Dis. 2018 Jun 20;77(9):1372–80. doi: 10.1136/annrheumdis-2018-212964 (PMC6104679; doi:10.1136/annrheumdis-2018-212964)
Supplement: Supplementary data [file annrheumdis-2018-212964supp001.docx]

Supplementary methods

**Materials:** Heparintinase I was from Calbiochem and chondroitinase ABC was Seikagaku Corp. Antibodies to perlecan (MAB1948) were from Chemicon; CTGF (sc-14939) and LTBP1 (sc-271140) were from Santa Cruz; TGFβ1,2,3 (neutralising) (MAB1835) and LAP1 (AB-246-NA) were from R&D systems; Type VI collagen (ab6588) and betaglycan (ab18885) were from Abcam; integrin β1 (anti-CD29, 102209), β3 (CD61, 104309) and αV (CD 51, 327904) from Biolegend; TGFβ1,2,3 (3711), phosphorylated SMAD2 (pSMAD2) (138D4), phosphorylated SMAD1/5 (pSMAD1/5) (41D10) and SMAD2 (86F7) were from cell signalling, All Alexa abs were from Invitrogen. Recombinant human TGFβ1 (112 aa) and BMP2 (115aa) were from Peprotech. Recombinant human activin A, ALK4/5 inhibitor (SB431542) and soluble human betaglycan (TGFβR3) were from R&D systems. Perlecan was purified from porcine knee articular cartilage as described previously {Vincent:2007dz}.

**Recombinant human CTGF protein expression and purification:** His-tagged CTGF construct was subcloned from a pcDNA3.1-*CTGF* vector (from Prof. Roger Mason, London) into a pCEP4 expression vector, which was then stably transfected into HEK293-EBNA cells (Invitrogen). The secreted fraction was purified by nickel affinity chromatography (GE Healthcare Ltd).

**Confocal microscopy:** Human articular cartilage explants were snap frozen in liquid nitrogen and embedded in OCT (Sakura). 7µm cryostat sections were cut. Sections were fixed in 100% methanol, enzymatic treated with chondroitinase ABC for 2hr at 37°C in PBS, blocked with 10% goat or donkey serum (Dako) for 1hr and then incubated with primary antibodies overnight. Sections were washed then incubated with secondary antibodies conjugated with Alexa Fluor 488 or Alexa Fluor 647, and propidium iodide.

**Cartilage and isolation of chondrocytes:**

Generation of porcine cartilage explants and isolation of porcine and human cells have been described by us previously [3][6][28]. Injury conditioned medium from porcine joints was generated by taking five 4mm diameter cartilage punch biopsies into 200 µl serum free medium. Each cartilage explant was cut in half twice (generating 4 pieces) to increase the ‘injury’ response. 50µl of each was added to the gel, thus the sample loading was controlled by tissue volume rather than total protein (as this would vary according to variables such as time from injury, period of conditioning etc). Murine hip cartilage was avulsed from the femoral head of 6-week-old mice. One hip was sufficient to generate 24h injury CM (50μl), two were required for 1h injury CM (50µl). Some hips were rested for 48h prior to generating a control or re-cut CM. The ear pinna was removed immediately post mortem and stripped of the overlying skin. Auricular cartilage mRNA was extracted as for murine hips. One ear was used for each experimental point.

**Induction of osteoarthritis in mice.** OA was induced in 10 week old male mice by surgical destabilisation of the medial meniscus (DMM) as previously described [8]. All in vivo experiments were approved by the UK Home Office (Project Licence holder, Vincent) and followed institutional ethical and procedural guide-lines. Histological sections from mice 8 and 12 weeks post surgery were scored according to a modified OARSI score where the three highest summed scores are added together to generate a total ‘summed’ score for each joint. Maximal cartilage thickness measurements from at least 5 sections (80μm apart) of each joint were averaged to give an average joint thickness measure.

**ELISA-based binding assay for CTGF and perlecan:** ELISA plates were coated with 0.05µg/well of purified perlecan in PBS for 3 hours at room temperature. Plates were washed, blocked with 1% BSA/PBS, then incubated with 0.05µg/well of CTGF in 100µl PBS. Plates were washed and incubated with primary antibody (perlecan or CTGF) in 1% BSA/PBS then secondary antibody for 1 hour at room temperature. After washing, TMB peroxidase substrate (TMB Microwell peroxidase substrate system, KPL, USA) was added. Fluorescence was detected by Spectramax fluorometer (Molecular Devices, USA). In some experiments plated perlecan was also treated in well with heparintinase or chondroitinase ABC (10mu/ml) for 2 hours at 37°C prior to incubation with CTGF.

**siRNA transfection:** Primary human articular chondrocytes (P1) were plated in 6 well plates at a density of 3 x 10^5^ cells/well (60% confluent) overnight in 10% FCS containing DMEM. Cells were transfected with Lipofectamine 2000 (Invitrogen) pre-incubated (20min) with respective siRNA oligos (CTGF: Hs01026927_g1 and Hs00170014_m1; LRP1: s8280 and s8279; TGFβR3: S24, S25 and S26)(Applied Biosystems), for 4hrs in Opti-Mem reduced serum medium (Gibco). Medium was replaced with normal 10% FCS containing DMEM for 48hr before starvation and stimulation.

**Microarray and RT-PCR**: Primary human chondrocytes were transfected with CTGF or scrambled siRNA and treated with recombinant CTGF (100ng/ml) for 8hr. RNA was extracted from the cells and purified using Qiagen RNeasy kit (Qiagen). The microarray was carried out using Illumina plate on a 2 HumanHT-12 v4 chip and scanned by BeadArray read (Illumina Inc.). Significantly regulated genes from the microarray were validated using TaqMan® RT PCR system (Applied Biosystems Inc.).

**Co-Immunoprecipitation:** Primary antibody (CTGF) or goat pre-immune IgG (1.5ng) were pre-incubated with protein G sepharose beads (30µl) (GE Healthcare). The IgG-beads were centrifuged and washed before adding 400µl of injury CM topped up to 1ml with PBS, and incubated overnight at 4°C. Immunoprecipitates were washed three times with cold PBS, separated by SDS-PAGE on precast 4-12% gels (Invitrogen), western blotted with antibodies against CTGF or LAP1.

**TGFβ1 ELISA assay:** 100μl of injury CM (24h) was assayed using the TGFβ1 Quantikine ELISA kit (MB100B from R&D Systems).. Briefly, 50μl of assay diluent was added to 50μl of injury CM generated from cut porcine cartilage. To distinguish active versus latent TGFβ the same samples were assayed after pre-treatment with HCl as per manufacturer’s protocol.
