# Supplementary figures and images for: Connective tissue growth factor contributes to joint homeostasis and osteoarthritis severity by controlling the matrix sequestration and activation of latent TGFβ
Source: Ann Rheum Dis. 2018 Jun 20;77(9):1372–80. doi: 10.1136/annrheumdis-2018-212964 (PMC6104679; doi:10.1136/annrheumdis-2018-212964)

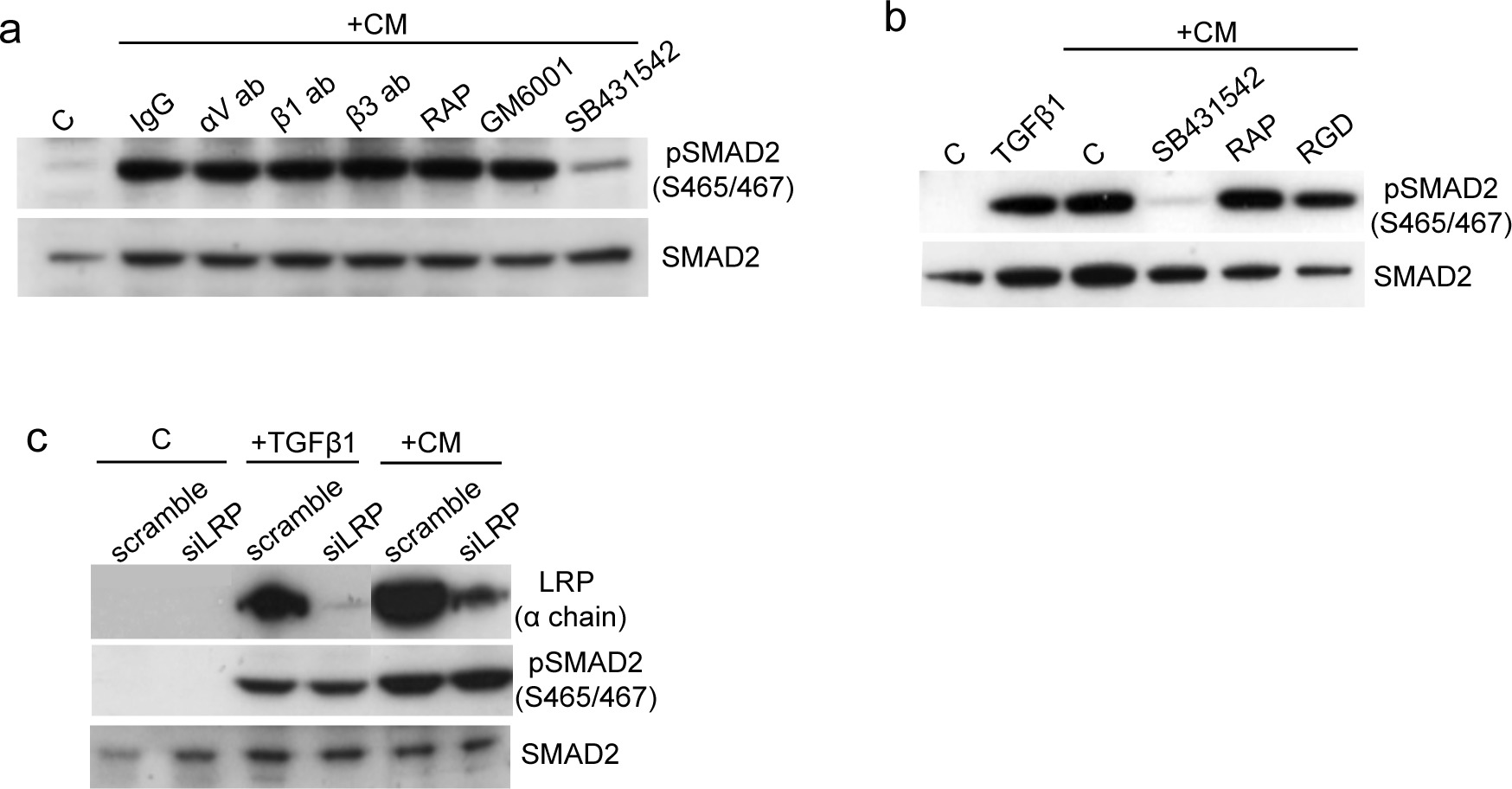

Supplement: Supplementary data [file annrheumdis-2018-212964supp003.jpg]

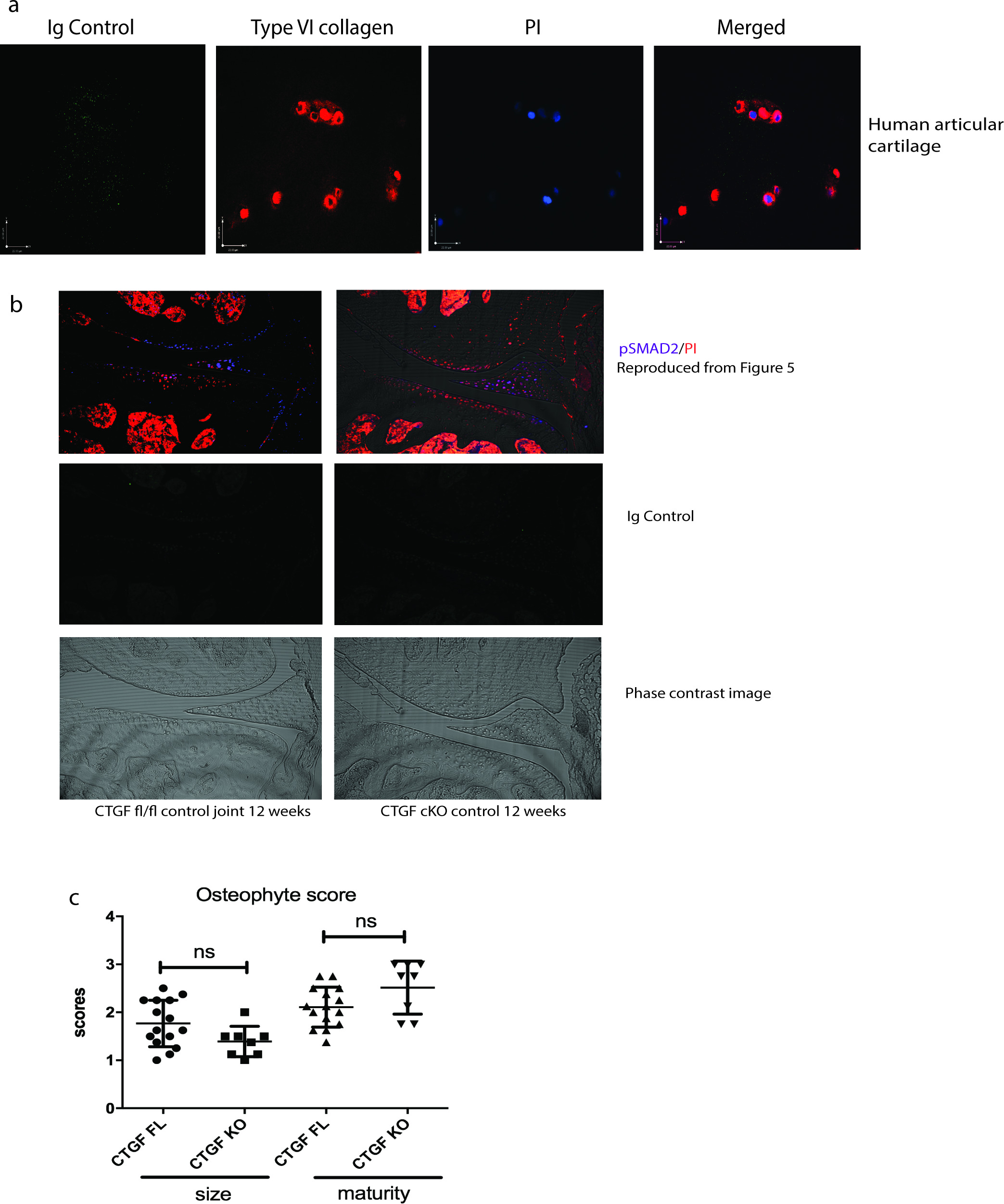

Supplement: Supplementary data [file annrheumdis-2018-212964supp004.jpg]
