# Supplementary material for: Connective tissue growth factor contributes to joint homeostasis and osteoarthritis severity by controlling the matrix sequestration and activation of latent TGFβ
Source: Ann Rheum Dis. 2018 Jun 20;77(9):1372–80. doi: 10.1136/annrheumdis-2018-212964 (PMC6104679; doi:10.1136/annrheumdis-2018-212964)
Supplement: Supplementary data [file annrheumdis-2018-212964supp002.docx]

|  | Auricular Cartilage | | | Whole Knee joint | | |
| --- | --- | --- | --- | --- | --- | --- |
| **Gene** | **Wild type (WT) mean**±SEM | **Knockout (relative to WT) mean**±SEM | **p value** | **Wild type (WT) mean**±SEM | **Knockout (relative to WT) mean**±SEM | **p value** |
|  | | | | | | |
| **Ctgf** | **1.018±0.115** | **0.028±0.005⇓** | **0.000** | **1.010±0.099** | **0.112±0.037⇓** | **0.001** |
| Tgfbr3 | 1.003±0.048 | 0.937±0.050 | 0.380 | 1.008±0.092 | 1.308±0.154 | 0.170 |
| Tgfbr2 | 1.004±0.053 | 0.976±0.022 | 0.641 | 1.001±0.036 | 1.282±0.192 | 0.225 |
| Tgfbr1 | 1.013±0.089 | 0.964±0.058 | 0.657 | **1.007±0.084** | **1.803±0.223** | **0.029** |
| Tgfbi | 1.021±0.119 | 0.916±0.066 | 0.473 | **1.001±0.037** | **1.501±0.103⇑** | **0.010** |
| Tgfb3 | 1.021±0.125 | 1.044±0.076 | 0.883 | 1.006±0.076 | 1.302±0.109 | 0.090 |
| Tgfb2 | 1.009±0.080 | 0.910±0.092 | 0.451 | **1.009±0.101** | **1.778±0.118⇑** | **0.008** |
| Tgfb1 | 1.014±0.105 | 0.932±0.111 | 0.609 | 1.038±0.198 | 0.823±0.056 | 0.356 |
| Sox9 | 1.013±0.095 | 0.881±0.099 | 0.379 | 1.030±0.170 | 1.359±0.324 | 0.419 |
| Smurf1 | 1.011±0.084 | 0.894±0.041 | 0.258 | **1.000±0.021** | **1.375±0.033⇑** | **0.000** |
| Runx2 | 1.010±0.082 | 0.732±0.087**⇓** | 0.059 | **1.003±0.054** | **1.573±0.170⇑** | **0.033** |
| Pmepa1 | 1.008±0.074 | 0.989±0.065 | 0.854 | **1.000±0.030** | **1.791±0.250⇑** | **0.036** |
| Nog | 1.036±0.164 | 1.005±0.143 | 0.894 | 1.001±0.024 | 1.323±0.262 | 0.288 |
| Ltbp4 | 1.015±0.100 | 1.065±1.060 | 0.708 | 1.010±0.102 | 0.825±0.025 | 0.154 |
| Ltbp3 | 1.017±0.105 | 0.929±0.044 | 0.467 | 1.010±0.096 | 1.336±0.204 | 0.222 |
| Ltbp2 | **1.012±0.089** | **0.786±0.027⇓** | **0.051** | 1.003±0.055 | 1.066±0.124 | 0.669 |
| Ltbp1 | 1.016±0.102 | 1.057±0.111 | 0.792 | 1.010±0.094 | 1.075±0.106 | 0.665 |
| Inhba | 1.012±0.085 | 1.141±0.079 | 0.312 | **1.034±0.195** | **1.615±0.074⇑** | **0.051** |
| Inha | 1.005±0.062 | 0.911±0.063 | 0.328 | 1.028±0.169 | 1.464±0.319 | 0.294 |
| Grem2 | 1.042±0.166 | 1.210±0.147 | 0.504 | 1.033±0.194 | 1.395±0.313 | 0.382 |
| GDF5 | 1.032±0.146 | 1.304±0.102 | 0.177 | 1.053±0.245 | 1.344±0.291 | 0.489 |
| FST | **1.007±0.066** | **0.798±0.039⇓** | **0.035** | 1.010±0.079 | 0.847±0.031 | 0.134 |
| Chrd | 1.010±0.076 | 0.855±0.067 | 0.179 | **1.00±0.0346** | **1.524±0.102⇑** | **0.008** |
| Bmpr2 | 1.013±0.093 | 0.971±0.018 | 0.676 | **1.000±0.031** | **1.454±0.106⇑** | **0.015** |
| Bmpr1b | 1.004±0.051 | 0.976±0.128 | 0.845 | 1.049±0.227 | 1.051±0.100 | 0.993 |
| Bmpr1a | 1.006±0.060 | 0.956±0.052 | 0.556 | **1.000±0.010** | **1.313±0.075⇑** | **0.014** |
| BMP7 | 1.008±0.072 | 0.994±0.151 | 0.936 | **1.012±0.110** | **1.946±0.257⇑** | **0.029** |
| BMP6 | **1.012±0.095** | **0.672±0.057⇓** | **0.022** | 1.012±0.111 | 1.094±0.142 | 0.670 |
| BMP4 | 1.004±0.050 | 0.923±0.043 | 0.264 | 1.004±0.061 | 1.127±0.085 | 0.305 |
| BMP2 | 1.018±0.108 | 0.968±0.048 | 0.689 | 1.002±0.046 | 0.983±0.135 | 0.900 |
| Acvrl1 | 1.006±0.062 | 0.847±0.062 | 0.123 | **1.013±0.110** | **1.653±0.050⇑** | **0.006** |
| Acvr2a | 1.007±0.071 | 0.927±0.053 | 0.401 | 1.008±0.088 | 1.178±0.017 | 0.131 |
| Acvr1 | 1.000±0.017 | 0.907±0.068 | 0.230 | 1.002±0.045 | 1.481±0.177**⇑** | 0.059 |
| ACAN | **1.000±0.020** | **0.722±0.096⇓** | **0.029** |  |  |  |
| Col2a1 | **1.037±0.154** | **2.984±0.533⇑** | **0.013** |  |  |  |
| Col1 | 1.033±0.155 | 0.795±0.117 | 0.267 |  |  |  |
| Col10 | 1.037±0.167 | 0.509±0.152⇓ | 0.058 |  |  |  |

**Supplementary Table 1. TGF related genes in auricular cartilage and whole joints of wild type and Ctgf^cKO^ mice 2 weeks after tamoxifen treatment.** Tgfbi-TGFb induced; Smurf-Smad Ubiquitination regulatory factor; Pmepa1-prostate transmembrane protein androgen induced; Nog-Noggin; Inhba-inhibin bA (dimer forms activin A); inha-inhibin alpha (heterodimer forms inhibins); Grem2-gremlin 2; GDF5-growth and differentiation factor 5; FST-follistatin (inhibitor of activin); Chrd-chordin; Acvrl-activin receptor like. Gene were expressed relative to 18S and normalised to the WT levels.
